# The Clinical Influence of Autophagy-Associated Proteins on Human Lung Cancer

**DOI:** 10.1155/2018/8314963

**Published:** 2018-01-09

**Authors:** Yuan Chen, Kai Leonie Schnitzler, Yunxia Ma, Miljana Nenkov, Bernhard Theis, Iver Petersen

**Affiliations:** Institute of Pathology, University Hospital Jena, Friedrich Schiller University Jena, Jena, Germany

## Abstract

Exploitation of autophagy might potentially improve therapeutic strategy. Here, we analyzed the protein expression of autophagy-associated genes including LC3A, LC3B, Beclin-1, p62, and Atg5 in 88–131 primary lung tumors by immunohistochemistry (IHC) on tissue-microarrays (TMAs). Additionally, the DNA methylation pattern of LC3A was investigated by bisulfite sequencing (BS) and methylation-specific-PCR (MSP). It turned out that the higher expression of LC3A protein was associated with adenocarcinoma compared to squamous cell carcinoma of lung (*p* = 0.008), positive staining of LC3B was significantly related to tumor grade (*p* = 0.006), and the protein expression of Beclin-1 was significantly correlated to pN stage (*p* = 0.041). The expression of p62 and Atg5 was however not significantly associated with any clinicopathological parameters. Downregulation of LC3A was related to DNA methylation in lung cancer cell lines, while in primary lung tumor samples, protein expression of LC3A was not significantly correlated with DNA methylation, and the methylation status of LC3A was not related to clinicopathological features. Taken together, our results suggest that autophagy-associated proteins such as LC3A, LC3B, and Beclin-1 might be potential biomarkers for subclassification, differentiation, and local metastasis in primary lung tumor, and epigenetic mechanism is partially responsible for gene silencing of LC3A in lung cancer cell lines.

## 1. Introduction

Lung cancer is the first leading cause of cancer-related death in both men and women in the USA [1]. In Germany, about 52,000 individuals were initially diagnosed with lung cancer in 2010 and about 43,000 individuals died of this disease [2]. Lung cancer is usually detected in late stage with local invasion and distant metastasis; together with therapy resistance, the prognosis for patients remains very poor with an overall 5-year survival rate of 16% for all stages in Europe and in the USA [3]. To achieve an early diagnosis and accurate evaluation of the prognosis, a better understanding of molecular mechanisms underlying lung cancer and identification of reliable biomarkers are therefore essential.

Autophagy is a genetically conserved cellular process that is important for the maintenance of intracellular metabolic homeostasis in yeast, plants, and animals [4]. It is a self-degradation system of cellular components through an autophagosomal-lysosomal pathway [5]. There are three distinct forms of autophagy described: macroautophagy, microautophagy, and chaperone-mediated autophagy. Autophagosome formation contains four main steps, namely initiation of autophagosomal formation, nucleation of autophagosomal vesicles, elongation of autophagosomal membrane, and recognition of cargos [6]. Initiation of autophagy by starvation or depletion of amino acids can be regulated by mammalian target of rapamycin (mTOR) unc-51 like autophagy activating kinase 1 (ULK1) Atg13 signaling [7]. Nucleation of autophagosomal vesicles can be regulated by the Beclin-1/vacuolar protein sorting 34 (VPS34)/VPS15/Atg14 complex [8, 9]. Two ubiquitin-like conjugation systems including complex of autophagy-associated genes (Atgs) Atg5/Atg12/Atg16 and microtubule-associated protein 1A/1B-light chain 3- (LC3-) II (converted form of LC3-I) regulate the elongation of the autophagosomal membrane [10, 11]. In the step of cargo recognition, p62/sequestosome 1 (SQSTM1) binds to both ubiquitinated proteins and LC3-II [12].

It has not yet been fully understood whether autophagy is a tumor suppressive or tumor promoting process. The apparent contradiction might be caused by the diverse effects of autophagy on tumor growth [13]. Accumulating evidence reveals that dysregulation of autophagy-associated proteins may lead to development of different tumors. For example, Beclin 1 expression is often downregulated in human breast cancer and *Beclin-1^+/−^* mice were found to be tumor prone indicating that Beclin-1 is a haploinsufficient tumor suppressor gene [14, 15]; LC3 was found to be highly expressed in gastrointestinal cancers [16]; *Atg7* liver conditional knockout mice developed hepatomegaly, a condition that may lead to malignant transformation [17]; and p62 was considered to be an oncogenic protein since its overexpression *in vivo* in the liver was sufficient to induce hepatocellular carcinoma without carcinogen administration [18].

It has been believed that epigenetically mediated aberrant gene silencing is closely related to pathogenesis of cancers including lung cancer. DNA methylation of autophagy genes plays an important role in cancer development. In glioblastoma, the most aggressive type of brain cancer, methylation of an upstream autophagy inducer ULK2 was found to be essential for astrocyte transformation and tumor growth [19]. In breast cancer, decreased expression of autophagy gene GABARAPL1 was associated with both DNA methylation and histone deacetylation [20], and methylation of Atg16L2 was found in patients with leukemia [21]. However, the issue of epigenetic regulation of autophagy-related genes has not been well addressed in human lung cancer [22].

In this study, we analyzed the clinical influence of five autophagy-related genes including LC3A, LC3B, Beclin-1, Atg5, and p62 and investigated the epigenetic regulation of one of the autophagy genes LC3A in human lung cancer.

## 2. Materials and Methods

### 2.1. Cell Lines, Cell Culture, and Drug Treatment

Human bronchial epithelial cells (HBECs) were obtained from Clonetics (San Diego, CA, USA) and cultured in BEG media (Lonza, Walkersville, USA). Five non-small-cell lung cancer (NSCLC) cell lines including H322, H1650, H1975, H2170, and H226 as well as one small cell lung cancer (SCLC) cell line COLO677 were purchased from the American Type Culture Collection (ATCC, Rockville, USA). Cells were grown in RPMI 1640 medium (Biochrom AG, Berlin, Germany) supplemented with 10% fetal bovine serum (FBS) (Biochrom) and maintained in a humidified atmosphere with 5% CO_2_ at 37°C.

For demethylation test, 5 *μ*M 5-aza-2′-deoxycytidine (5-Aza) (Sigma Chemical Co., St. Louis, MO USA) was added to the medium on days 0, 2, and 4 when cell lines (H322, H1650, H1975, H226, and COLO677) reached 50% confluence. Then, cells were harvested on day 4 for RNA isolation.

### 2.2. RNA Isolation and Real-Time RT-PCR

Total RNA was extracted from cells using the Trizol reagent (peqGOLD TriFastTM, VWR, Germany) according to the manufacturer's instructions. One hundred nanograms of total RNA were reversely transcribed into cDNA using a QuantiTect Reverse Transcription Kit (Qiagen, Hilden, Germany).

Real-time RT-PCR was performed in 0.1 ml tubes on the Rotor-Gene 6000 (Qiagen, Hilden, Germany) in the presence of the FastStart Universal SYBR Green Master (Roche, Mannheim, Germany). Twenty-five nanograms of RNA were applied for PCR amplification under the following conditions: 95°C 15 min and 95°C 15 sec and 60°C 30 sec for 45 cycles. Glyceraldehyde-3-phosphate dehydrogenase (GAPDH) was used as control. Primer sequences are shown in Supplementary Data 1.

### 2.3. Genomic DNA Isolation, Bisulfite Modification, Bisulfite Sequencing, and Methylation-Specific PCR

Genomic DNA from cell lines and patient samples was isolated using a QIAamp FFPE kit (Qiagen) according to the manufacturer's recommendations. Manual microdissection was carried out before DNA extraction. Bisulfite modification of genomic DNA was performed by using an EZ DNA Methylation kit (Zymo Research, Freiburg, Germany).

Bisulfite-treated genomic DNA was subjected to PCR amplification. Two pairs of primer (Supplementary Data 1) from promoter region and exon 4 of the LC3A gene were designed to amplify bisulfite-modified DNA. PCR products were purified using a DNA Clean & Concentrator Kit (Zymo Research) and sequenced by capillary electrophoresis (LGC, Berlin, Germany).

Methylation-specific PCR (MSP) was carried out as described previously [23]. Primer sequences are listed in Supplementary Data 1. The conditions for MSP-PCR amplification are as follows: 95°C 15 min; 94°C 1 min, 58°C 30 s, and 72°C 30 s for 35 cycles; and 72°C 10 min. Each of the PCR amplifications was repeated at least once to confirm the results.

### 2.4. Patient Samples, Tissue Microarray (TMA) Construction, and Immunohistochemistry (IHC)

In total, two lung tumor TMAs were applied for IHC with one containing 88 samples from University Hospital Jena and the other containing 131 samples from University Hospital Charité. All of the patients were undergoing surgical operation at the Department of Surgery of these two hospitals from 1995 to 2010. No adjuvant radiotherapy or chemotherapy was administered before surgery. The clinicopathological data are based on the original tumor not based on tumor TMAs according to the WHO classification of lung cancer 2004. The study was approved by the local ethical committee (Nr. 3815-07/13).

Tissue microarrays (TMAs) were constructed using a manual tissue arrayer purchased from Beecher Instruments (Woodland, WI, USA) as previously described [24]. Two tissue cylinders per tumor with a diameter of 0.6 mm were present in the TMAs.

Immunohistochemistry (IHC) was performed. Briefly, 3 *μ*m sections from the TMAs were dewaxed with xylene and gradually hydrated. Antigen retrieval was performed by treatment in a pressure cooker for 6 min.

Primary antibodies against autophagy-associated proteins including LC3A, LC3B, Beclin-1, p62, and Atg5 (Supplementary Data 2) were incubated at room temperature for 1 h. Detection was carried out according to the manufacturer's instructions (LSAB-2 kits, DAKO, Germany). All the IHC slides were evaluated by a pathologist (B. Theis) who was blinded to the clinical information. IHC was scored semiquantitatively as negative (<10% positively stained cells; score 0), weak (10–25% positively stained cells; score 1), moderate (26–50% positively stained cells; score 2), or strong (more than 50% positively stained cells; score 3). For statistical analysis, scores 0 and 1 together were considered low expression, while scores 2 and 3 together were considered high expression.

### 2.5. Statistical Analysis

Statistical analysis was performed by using SPSS19.0 software. To compare protein expression or methylation status of LC3A with clinicopathological features, chi-square test (*χ* ^2^) was applied. For pairwise comparisons, Pearson correlation analysis was performed. Kaplan–Meier survival curves were constructed for statistical significance with the log rank tests. Student's *t*-test was applied to compare the gene expression levels. A *p* value < 0.05 was considered statistically significant.

## 3. Results

### 3.1. Expression of LC3A mRNA in Lung Cancer Cell Lines and Demethylation Test

**Figure 1:**
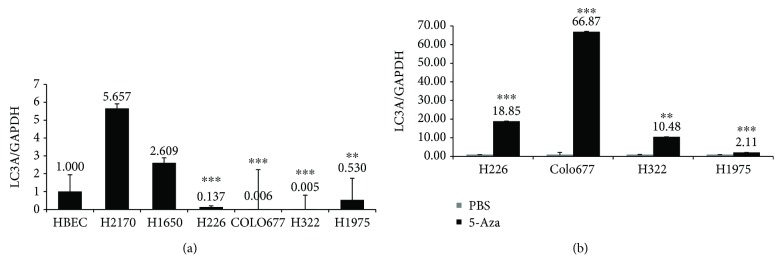
(a) LC3A mRNA expression analysis in lung cancer cell lines and human bronchial epithelial cells (HBEC). LC3A mRNA expression was analyzed by real-time RT-PCR, showing that LC3A expression was increased in lung cancer cell lines H2170 and H1650 and downregulated in H226, COLO677, H322, and H1975 compared to HBEC. Relative LC3A mRNA expression was normalized to GAPDH mRNA expression. (b) Demethylation tests in lung cancer cell lines. Real-time RT-PCR showed that after treatment with 5 *μ*M of 5-aza-2′-deoxycytidine (5-Aza) for 96 h, LC3A mRNA expression was heterogeneously upregulated in the 4 cell lines. Student's *t*-test was applied to compare the gene expression levels. ^∗∗^ *p* < 0.01; ^∗∗∗^ *p* < 0.001.

Compared to normal bronchial epithelial cells (HBEC), the expression of LC3A was downregulated in lung cancer cell lines H226, COLO677, H322, and H1975 at mRNA level, while in the cell lines H2170 and H1650, the expression of LC3A was higher than in HBEC (Figure 1(a)). In the study by Nihira et al., they found the downregulation of LC3A at both mRNA and protein levels [22].

Since epigenetic mechanism could be responsible for gene silencing, we wanted to know whether downregulation of LC3A in cell lines H322, H1975, H226, and Colo677 was due to DNA hypermethylation. For this purpose, cells were treated with the demethylation reagent 5-Aza. Real-time RT-PCR showed that in the cell lines H322, H226, and COLO677, the expression of LC3A mRNA was restored after 5-Aza modification. By contrast, only a slight upregulation of LC3A was observed in H1975 (Figure 1(b)). These results suggest that DNA hypermethylation could be partially responsible for gene silencing of LC3A, and other mechanisms might also be involved.

### 3.2. Analysis of Methylation Status of LC3A by Bisulfite Sequencing and Methylation-Specific PCR

To further analyze the methylation status of LC3A in lung cancer cell lines and primary lung tumor samples, bisulfite sequencing (BS) and methylation-specific PCR (MSP) were carried out.

**Figure 2:**
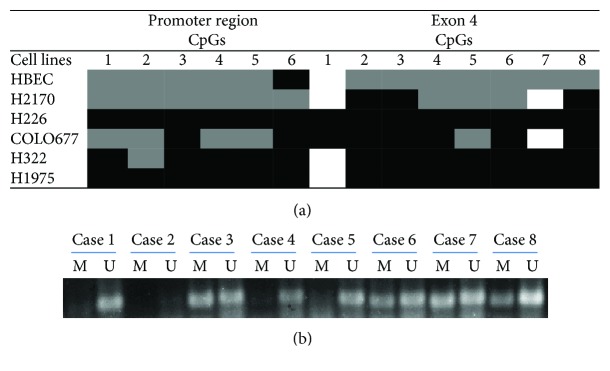
(a) Methylation status of LC3A in 5 lung cancer cell lines and HBEC. Bisulfite sequencing (BS) showed a heterogeneous methylation pattern in the promoter region and exon 4 of the LC3A gene in lung cancer cell lines. White: unmethylated; grey: partially methylated or only one allele is methylated; black: totally methylated or two alleles are methylated. 1–6: 6 CpG sites in promoter region; 1–8: 8 CpG sites in exon 4. (b) Representative results from methylation-specific PCR (MSP) showing that LC3A was unmethylated in cases 1, 4, and 5 while partially methylated in cases 3, 6, 7, and 8. Case 2 was excluded for the statistical analysis (see Supplementary Table 3), since no PCR products were observed most probably due to degraded genomic DNA. U: unmethylated; M: methylated.

The methylation status of LC3A in the promoter region and exon 4 including 6 and 8 CpG dinucleotides, respectively, was analyzed in cancer cell lines H226, COLO677, H322, and H1975 as well as in normal cells HBEC by BS (Figure 2). In general, the expression of LC3A correlates well with the methylation status in both regions. HBEC cells with medium expression of LC3A are partially methylated. H266 and H1975 have weak LC3A expression and both cell lines are completely methylated in promoter region and exon 4. COLO677 exhibits no LC3A expression and is highly methylated in exon 4 and partially methylated in promoter region. Furthermore, H322 without endogenous expression of LC3A was found to be completely methylated in exon 4 and almost completely methylated in promoter region.

In primary lung tumor samples, methylation-specific PCR was performed to analyze the LC3A methylation status. Since primers designated in the promoter region or exon 4 resulted in unspecific PCR products of MSP amplification (data not shown), we analyzed the methylation status of LC3A in exon 3 using primer designed to amplify 10 CpG dinucleotides. Representative results from MSP are shown in Figure 2(b). It turned out that 36 out of 56 samples (62.3%) were methylated, and among the positive ones, the majority samples were partially methylated. The methylation status was not significantly associated with clinicopathological parameters including tumor subtype, stage, and grading (Supplementary Data 3). The methylation status was also not significantly related to protein expression of LC3A (data not shown).

### 3.3. Clinical Significance of Autophagy-Related Proteins

We performed immunohistochemistry (IHC) on tissue-microarray (TMA) to evaluate the clinical relevance of autophagy-related proteins including LC3A, LC3B, Beclin-1, Atg5, and p62 in primary lung tumor.

**Figure 3:**
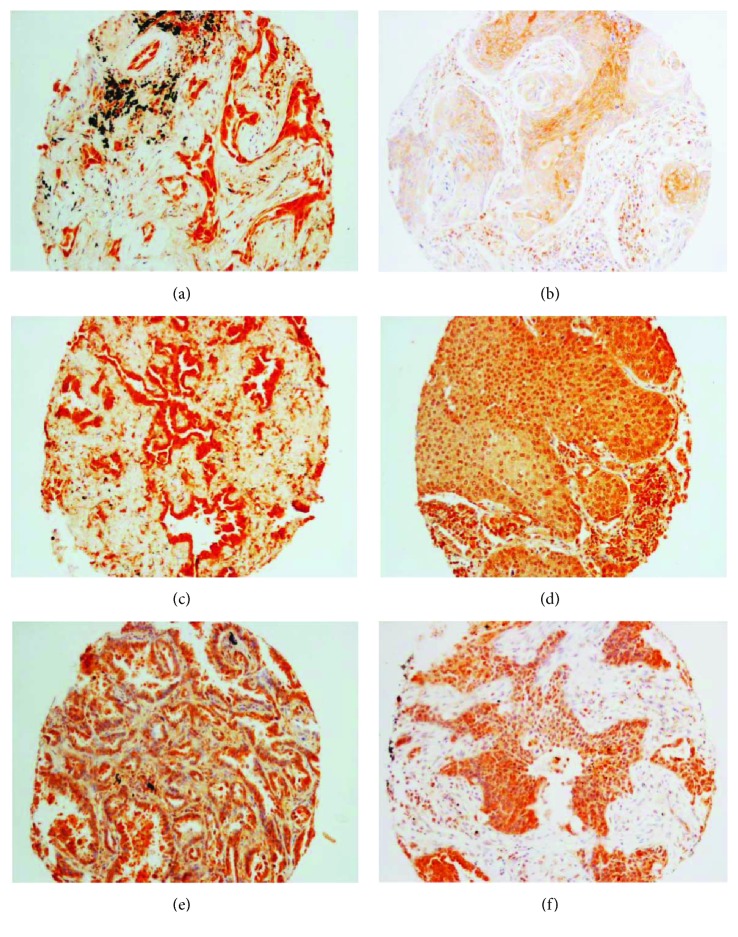
Representative images for the expression of autophagy-associated proteins in primary lung cancer. (a) LC3A protein expression in lung adenocarcinoma (ADC). (b) LC3A protein expression in lung squamous cell carcinoma (SCC). (c) LC3B protein expression in lung ADC. (d) LC3B protein expression in lung SCC. (e) Beclin-1 protein expression in lung ADC. (f) Beclin-1 expression in lung SCC.

LC3A, also called MAP1LC3A (microtubule-associated proteins 1A/1B light chain 3A), one of the mammalian homologues of yeast autophagy-related gene 8 (Atg8), was found to be expressed in 27 out of 88 (30.7%) samples. A diffuse cytoplasmic staining pattern could be observed (Figures 3(a) and 3(b)). Stroma cells and lymph cells were unstained with LC3A antibody. Statistical analysis revealed that the higher expression of LC3A was associated with tumor subtype: adenocarcinoma of lung exhibited significantly more expression of LC3A in comparison to lung squamous cell carcinoma (SCC) (*p* = 0.008; Table 1).

LC3B (microtubule-associated proteins 1A/1B light chain 3B), another human LC3 gene family member, was stained positive in 63 out of 117 (53.8%) samples. Similar to LC3A, diffuse cytoplasmic staining pattern was found in LC3B-positive tumors (Figures 3(c) and 3(d)). The expression of LC3B is significantly related to tumor grade: tumors with higher grades or more poorly differentiated tumors had higher expression of LC3B (*p* = 0.003; Table 2). However, caution should be taken that the dynamic process of the conversion from LC3 I to LC3 II, an indicator, or autophagy cannot be accessed by IHC.

Beclin-1 (also called autophagy-related gene 6 or Atg6) positive staining was found in 46 out of 123 (37.4%) tumor samples. Diffuse cytoplasmic staining was considered positive (Figures 3(e) and 3(f)). Connective tissues were unstained, while inflammatory cells were partially positively stained (data not shown). The expression levels were significantly correlated to pN stage (*p* = 0.041): patients whose tumors had higher expression of Beclin-1 were more likely to get a lymph node metastasis (Table 3).

The protein expression of p62 (also called sequestosome-1) and Atg5 (autophagy protein 5) was found in 44 out of 118 (37.3%) and 70 out of 131 (53.4%) patients with primary lung cancer, respectively. However, the protein staining was not significantly linked to any clinicopathological parameters including age, gender, tumor subtype, tumor grade, and stage (Supplementary Data 4 and 5).

Overall comparison of the protein expression was calculated by the pairwise correlation coefficient from one sample to another. It turned out that the p62 expression was significantly associated with the expression of Beclin-1 and LC3B (Table 4; *p* < 0.01). The same TMA was used to analyze the expression of these three proteins. Similarly, the LC3A expression was significantly related to the expression of Atg5 based on the data analysis from the second TMA (Table 5; *p* < 0.01).

We further analyzed the impact of these five autophagy-associated proteins on clinical outcome by Kaplan–Meier analysis. However, statistical analysis did not reveal any significance. It seems that none of the proteins might be a potential prognostic marker for patients with primary lung cancer (data not shown).

## 4. Discussion

The role of autophagy in tumor is controversial, since it can exert multifactorial influence on tumorigenesis, tumor progression, cancer therapeutics, and prevention dependent on cellular context [25]. Here, we demonstrated the epigenetic regulation and clinical influence of autophagy-related proteins on human lung cancer.

LC3A and LC3B are structural proteins of autophagosomal membranes which have been proposed as markers for detection of cells undergoing autophagy [26]. LC3A, not LC3B, was found to be frequently downregulated in various cancer cell lines, and gene silencing of LC3A could be explained by DNA methylation in esophageal squamous cell carcinoma and lung cancer [22, 27]. In line with this, we found decreased expression of LC3A in 4 out of 6 lung cancer cell lines compared to normal human bronchial epithelial cells (HBEC), which correlated to DNA methylation of LC3A in promoter region and exon 4. In primary lung cancer, the majority samples (36 out of 56) exhibited partial methylation of LC3A; however, the methylation status of LC3A seems to have no clinical significance in lung cancer.

Three different staining patterns of LC3A protein were detected in human cancer cells, namely, diffuse cytoplasmic, cytoplasmic/perinuclear, and “stone-like” structures (SLS) [28–30]. In the analysis of LC3A protein expression, we found the diffuse cytoplasmic distribution of LC3A, and the positive staining of LC3A was significantly associated with adenocarcinoma of lung. In the study by Nihira et al., a cytoplasmic staining of LC3A was found in more than half of the lung ADC samples [22]. In skin cancer including cutaneous squamous cell carcinoma and malignant melanoma, SLS-staining of LC3A was associated with tumor hypoxia and aggressiveness [28, 30]. In colorectal carcinoma, perinuclear LC3A accumulation indicated good prognosis, while increased numbers of SLS was linked to metastasis and poor prognosis [31].

LC3B, a microtubule-associated protein, has been considered a marker for autophagy, since cytosolic LC3B-I protein is converted into LC3B-II and binds to autophagosomes when autophagy occurs [32]. Overexpression of LC3B correlates with malignant progression and aggressive behavior and predicts a poor clinical outcome in hepatocellular carcinoma, glioblastoma, and pancreatic ductal adenocarcinoma [33–35]. In line with the study by Zheng et al. which revealed that higher expression of LC3B was related to more poorly differentiated colorectal carcinoma [36], we found that increased expression of cytoplasmic LC3B is significantly related to higher tumor grade, suggesting an important role that LC3B may play in lung tumor differentiation. It is also worth mentioning that the reliability and specificity of the LC3B antibody applied in our study has already been tested in FFPE samples before [37]. Additionally, it was reported that LC3B was regulated by the transcription factor Egr-1 in cigarette smoke-induced chronic obstructive pulmonary disease [38].

Beclin-1, an interactor of the oncogenic antiapoptotic protein BCL-2, interacts with PI3-kinase class III to trigger autophagy [39]. These biochemical properties suggest that Beclin-1 is involved in two fundamentally important cell biological pathways: autophagy and apoptosis. Beclin-1 has been considered a potential prognostic biomarker in several types of cancer including salivary gland adenoid cystic cancer, ovarian cancer, and lung cancer [40–42]. However, in our study, we did not find a significant impact of Beclin-1 on patients' survival most probably due to the small size of samples investigated. In line with the role of Beclin-1 in gastric cancer [43], but contrast to its role in papillary thyroid cancer [44], we found that the lower expression of Beclin-1 was significantly linked to less lymph node metastasis.

p62, also known as sequestosome-1, is one of the factors that target specific cargoes for autophagy. In breast cancer, p62 expression was highest in HER2-positive tumors [45]. In gastric cancer, overexpression of p62 was related to tumor metastasis [46]. In lung cancer, p62 expression was found to be negatively correlated with TNM stage and lymph node metastasis [47]. In our study, however, no clinical significance of p62 was found when only the cytoplasmic staining was considered positive for analysis. It is not yet clear if the nuclear staining of p62 could really reflect the status of autophagy [37].

Atg5, an E3 ubiquitin ligase, is proposed to be necessary for autophagy due to its role in autophagosome elongation [11]. Expression of Atg5 could predict favorable disease-free survival in patients with breast cancer [48]. Somatic mutations and downregulation of Atg5 were found in gastrointestinal cancer [49]. In lung cancer, we found that more than 40% of samples (61 out of 131) had low expression of Atg5 protein and tumors with higher expression of Atg5 usually were well differentiated; however, this correlation has not yet reached statistical significance (*p* = 0.069). For each protein, sample size applied for the investigation is different since slides cut from TMA block contain a different number of missing cores, and for LC3A, only one TMA block (samples from Charité) was available.

Taken together, the analysis of autophagy-related genes indicates a complex and not yet fully understood role of autophagy in cancer pathogenesis. This supports the notion that autophagy is a double-edged sword in cancer. On the one hand, it could be tumor-suppressive due to its role in the removal of damaged organelles and toxic protein aggregates to prevent genome instability [50], and on the other hand, it may enhance tumor cell survival and contribute to therapy resistance. There are probably many other cellular mechanisms linked to autophagy being also relevant to tumor biology like hypoxia, inflammation, and immunity. Since autophagy is fundamental for hemostasis and cellular interaction and it is potentially druggable, the analysis of its constituents in lung cancer merits further studies.

## Figures and Tables

**Table 1:** Correlation between LC3A and clinicopathological parameters in primary lung tumor.

		LC3A		*p* value
		Low	High	
Type	ADC	24	21	**0.008**
	SCC	30	6	
Gender	Male	48	19	0.425
	Female	13	8	
Age	≤65	39	21	0.318
	>65	21	6	
pN	0-1	39	20	0.798
	>1	17	7	
pT	0-1	10	10	0.057
	>1	48	17	
Grade	1-2	32	15	1
	>2	29	13	

ADC: adenocarcinoma; SCC: squamous cell carcinoma.

**Table 2:** Correlation between LC3B and clinicopathological parameters in primary lung tumor.

		LC3B		*p* value
		Low	High	
Type	ADC	13	9	0.250
	SCC	39	47	
Gender	Male	46	55	0.740
	Female	8	8	
pT	0-1	19	19	0.563
	>1	35	44	
pN	0-1	49	54	0.569
	>1	5	9	
Grade	1-2	18	7	**0.003**
	>2	36	56	

**Table 3:** Correlation between Beclin-1 and clinicopathological parameters in primary lung tumor.

		Beclin-1		*p* value
		Low	High	
Type	ADC	14	10	0.815
	SCC	56	34	
Gender	Male	67	40	1
	Female	10	6	
pT	0-1	62	35	0.649
	>1	15	11	
pN	0-1	47	19	**0.041**
	>1	30	27	
Grade	1-2	51	26	0.337
	>2	26	46	

**Table 4:** Pearson correlation between p62, Beclin1, and LC3B.

	p62	Beclin 1	LC3B
p62	1.000		
Beclin 1	0.290 (*p* < 0.01)	1.000	
LC3B	0.256 (*p* < 0.01)	0.188	1.000

**Table 5:** Pearson correlation coefficient between LC3A and ATG5.

	LC3A	ATG5
LC3A	1.000	
ATG5	0.300 (*p* < 0.05)	1.000

## Supplementary Materials

Supplementary Materials Table S1: sequences of primers for LC3A. Table S2: antibodies used for immunohistochemistry (IHC). Table S3: correlation between LC3A methylation and clinicopathological parameters in primary lung tumor. Table S4: correlation between p62 protein expression and clinicopathological parameters in primary lung tumor. Table S5: correlation between Atg5 protein expression and clinicopathological parameters in primary lung tumor.

## References

[1] Siegel R., Miller K., Jemal A. (2015). Cancer statistics, 2015. *CA: a Cancer Journal for Clinicians*.

[2] Robert Koch Institute and the Association of Population-based Cancer Registries in Germany (2014). *Cancer in Germany 2009/2010*.

[3] Compelmann D., Eberhardt R., Herth F. J. (2011). Advanced malignant lung disease: what the specialist can offer. *Respiration*.

[4] Yang Z., Klionsk D. J. (2010). Eaten alive: a history of macroautophagy. *Nature Cell Biology*.

[5] Pyo J. O., Nah J., Jung Y. K. (2012). Molecules and their functions in autophagy. *Experimental & Molecular Medicine*.

[6] Zhang J. H. (2012). Teaching the basics of autophagy and mitophagy to redox biologists—mechanisms and experimental approaches. *Redox Biology*.

[7] Ganley I. G., Lam du H., Wang J., Ding X., Chen S., Jiang X. (2009). ULK1.ATG13.FIP200 complex mediates mTOR signaling and is essential for autophagy. *Journal of Biological Chemistry*.

[8] Mehrpour M., Esclatine A., Beau I., Codogno P. (2010). Overview of macroautophagy regulation in mammalian cells. *Cell Research*.

[9] Kim J., Kundu M., Viollet B., Guan K. L. (2011). AMPK and mTOR regulate autophagy through direct phosphorylation of Ulk. *Nature Cell Biology*.

[10] Tanida I., Ueno T., Kominami E. (2008). LC3 and autophagy. *Methods in Molecular Biology*.

[11] Walczak M., Martens S. (2013). Dissecting the role of the Atg12-Atg5-Atg16 complex during autophagosome formation. *Autophagy*.

[12] Feng Y., He D., Yao Z., Klionsky D. J. (2014). The machinery of macroautophagy. *Cell Research*.

[13] Nakahira K., Choi A. (2013). Autophagy: a potential therapeutic target in lung diseases. *American Journal of Physiology Lung Cellular and Molecular Physiology*.

[14] Liang X. H., Jackson S., Seaman M. (1999). Induction of autophagy and inhibition of tumorigenesis by *beclin 1*. *Nature*.

[15] Qu X., Yu J., Bhagat G. (2003). Promotion of tumorigenesis by heterozygous disruption of the beclin 1 autophagy gene. *Journal of Clinical Investigation*.

[16] Yoshioka A., Miyata H., Doki Y. (2008). LC3, an autophagosome marker, is highly expressed in gastrointestinal cancers. *International Journal of Oncology*.

[17] Komatsu M., Waguri S., Ueno T. (2005). Impairment of starvation-induced and constitutive autophagy in Atg7-deficient mice. *The Journal of Cell Biology*.

[18] Umemura A., He F., Taniguchi K. (2006). p62, upregulated during preneoplasia, induces hepatocellular carcinogenesis by maintaining survival of stressed HCC-initiating cells. *Cancer Cell*.

[19] Shukla S., Patric I. R., Patil V. (2014). Methylation silencing of ULK2, an autophagy gene, is essential for astrocyte transformation and tumor growth. *Journal of Biological Chemistry*.

[20] Hervouet E., Claude-Taupin A., Gauthier T. (2015). The autophagy GABARAPL1 gene is epigenetically regulated in breast cancer models. *BMC Cancer*.

[21] Dunwell T., Hesson L., Rauch T. A. (2010). A genome-wide screen identifies frequently methylated genes in haematological and epithelial cancers. *Molecular Cancer*.

[22] Nihira K., Miki Y., Iida S. (2014). An activation of LC3A-mediated autophagy contributes to de novo and acquired resistance to EGFR tyrosine kinase inhibitors in lung adenocarcinoma. *The Journal of Pathology*.

[23] Chen Y., Yang L., Cui T., Pacyna-Gengelbach M., Petersen I. (2015). HOPX is methylated and exerts tumour-suppressive function through Ras-induced senescence in human lung cancer. *The Journal of Pathology*.

[24] Cui T., Chen Y., Yang L. (2012). The p53 target gene desmocollin 3 acts as a novel tumour suppressor through inhibiting EGFR/ERK pathway in human lung cancer. *Carcinogenesis*.

[25] Dikic I., Johansen T., Kirkin V. (2010). Selective autophagy in cancer development and therapy. *Cancer Research*.

[26] Koukourakis M. I., Kalamida D., Giatromanolaki A. (2015). Autophagosome proteins LC3A, LC3B and LC3C have distinct subcellular distribution kinetics and expression in cancer cell lines. *PLoS One*.

[27] Bai H., Inoue J., Kawano T., Inazawa J. (2012). A transcriptional variant of the *LC3A* gene is involved in autophagy and frequently inactivated in human cancers. *Oncogene*.

[28] Sivridis E., Giatromanolaki A., Karpathiou G., Karpouzis A., Kouskoukis C., Koukourakis M. I. (2011). LC3A-positive “stone-like” structures in cutaneous squamous cell carcinomas. *The American Journal of Dermatopathology*.

[29] Sivridis E., Koukourakis M. I., Mendrinos S. E., Touloupidis S., Giatromanolaki A. (2013). Patterns of autophagy in urothelial cell carcinomas--the significance of “stone-like,” structures (SLS) in transurethral resection biopsies. *Urologic Oncology*.

[30] Sivridis E., Koukourakis M. I., Mendrinos S. E. (2011). Beclin-1 and LC3A expression in cutaneous malignant melanomas: a biphasic survival pattern for beclin-1. *Melanoma Research*.

[31] Giatromanolaki A., Koukourakis M. I., Harris A. L., Polychronidis A., Gatter K. C., Sivridis E. (2010). Prognostic relevance of light chain 3 (LC3A) autophagy patterns in colorectal adenocarcinomas. *Journal of Clinical Pathology*.

[32] Nakatogawa H., Ichimura Y., Ohsumi Y. (2007). Atg8, a ubiquitin-like protein required for autophagosome formation, mediates membrane tethering and hemifusion. *Cell*.

[33] Wu D. H., Jia C. C., Chen J. (2014). Autophagic LC3B overexpression correlates with malignant progression and predicts a poor prognosis in hepatocellular carcinoma. *Tumour Biology*.

[34] Giatromanolaki A., Sivridis E., Mitrakas A. (2014). Autophagy and lysosomal related protein expression patterns in human glioblastoma. *Cancer Biology & Therapy*.

[35] Ko Y. H., Cho Y. S., Won H. S. (2013). Prognostic significance of autophagy-related protein expression in resected pancreatic ductal adenocarcinoma. *Pancreas*.

[36] Zheng H. Y., Zhang X. Y., Wang X. F., Sun B. C. (2012). Autophagy enhances the aggressiveness of human colorectal cancer cells and their ability to adapt to apoptotic stimulus. *Cancer Biology & Medicine*.

[37] Schläfli A. M., Berezowska S., Adams O., Langer R., Tschan M. P. (2015). Reliable LC3 and p62 autophagy marker detection in formalin fixed paraffin embedded human tissue by immunohistochemistry. *European Journal of Histochemistry*.

[38] Chen Z. H., Kim H. P., Sciurba F. C. (2016). Egr-1 regulates autophagy in cigarette smoke-induced chronic obstructive pulmonary disease. *PLoS One*.

[39] Trincheri N. F., Follo C., Nicotra G., Peracchio C., Castino R., Isidoro C. (2008). Resveratrol-induced apoptosis depends on the lipid kinase activity of Vps34 and on the formation of autophagolysosomes. *Carcinogenesis*.

[40] Liang L. Z., Ma B., Liang Y. J. (2012). High expression of the autophagy gene Beclin-1 is associated with favorable prognosis for salivary gland adenoid cystic carcinoma. *Journal of Oral Pathology & Medicine*.

[41] Valente G., Morani F., Nicotra G. (2014). Expression and clinical significance of the autophagy proteins BECLIN 1 and LC3 in ovarian cancer. *BioMed Research International*.

[42] Zhou W., Yue C., Deng J. (2013). Autophagic protein Beclin 1 serves as an independent positive prognostic biomarker for non-small cell lung cancer. *PLoS One*.

[43] Chen Y. B., Hou J. H., Feng X. Y. (2012). Decreased expression of Beclin 1 correlates with a metastatic phenotypic feature and adverse prognosis of gastric carcinomas. *Journal of Surgical Oncology*.

[44] Li X., Xu H., Ma H. (2013). Beclin 1 is highly expressed in papillary thyroid carcinoma and correlates with lymph node metastasis. *Acta Chirurgica Belgica*.

[45] Choi J., Jung W., Koo J. S. (2013). Expression of autophagy-related markers beclin-1, light chain 3A, light chain 3B and p62 according to the molecular subtype of breast cancer. *Histopathology*.

[46] Masuda G. O., Yashiro M., Kitayama K. (2016). Clinicopathological correlations of autophagy-related proteins LC3, Beclin 1 and p62 in gastric cancer. *Anticancer Research*.

[47] Wang X., Du Z., Li L., Shi M., Yu Y. (2015). Beclin 1 and p62 expression in non-small cell lung cancer: relation with malignant behaviors and clinical outcome. *International Journal of Clinical & Experimental Pathology*.

[48] Wang L., Yao L., Zheng Y. Z. (2015). Expression of autophagy-related proteins ATG5 and FIP200 predicts favorable disease-free survival in patients with breast cancer. *Biochemical and Biophysical Research Communications*.

[49] An C. H., Kim M. S., Yoo N. J., Park S. W., Lee S. H. (2011). Mutational and expressional analyses of ATG5, an autophagy-related gene, in gastrointestinal cancers. *Pathology - Research and Practice*.

[50] Chen H. Y., White E. (2011). Role of autophagy in cancer prevention. *Cancer Prevention Research*.

