# Supplementary material for: The Clinical Influence of Autophagy-Associated Proteins on Human Lung Cancer
Source: Dis Markers. 2018 Jan 9;2018:8314963. doi: 10.1155/2018/8314963 (PMC5818951; doi:10.1155/2018/8314963)
Supplement: Supplementary Materials — Table S1: sequences of primers for LC3A. Table S2: antibodies used for immunohistochemistry (IHC). Table S3: correlation between LC3A methylation and clinicopathological parameters in primary lung tumor. Table S4: correlation between p62 protein expression and clinicopathological parameters in primary lung tumor. Table S5: correlation between Atg5 protein expression and clinicopathological parameters in primary lung tumor. [file 8314963.f1.pdf]

Table S1. Sequences of primers for LC3A

PCR	Primer name	Sequence (5'-3')
Real-time RT-PCR		
	LC3A-F	CCTGGACAAGACCAAGGTTTTTG
	LC3A-R	GTCTTTCTCCTGCTCGTAGATG
	GAPDH-F	GACAGTCAGCCGCATCTTCT
	GAPDH-R	TTAAAAGCAGCCCTGGTGAC
MSP in exon 3		
	MSP-F	ATCGGTTTTTAAGTAGCGGC
	MSP-R	ACCTAACGTCACGAAACTAACGT
	UMSP-F	AGATTGGTTTTTAAGTAGTGGTGG
	UMSP-R	AACCTAACATCACAAAACTAACATC
BS in promoter region		
	BS-F	TTGTTATTTTTGATTGTATGGAAGTTAG
	BS-R	CCTAAAACCAATCCTTCCACTATAC
BS in exon 4		
	BS-F	TTAGTATAGTATGGTGAGTGTGTTTA
	BS-R	CCCCTACTACTAACTCAAAAACC

BS: bisulfite sequencing; MSP: methylation-specific-PCR; UMSP: unmethylation-specific-PCR

Table S2. Antibodies used for immunohistochemistry (IHC)

Name	Host	Company	Dilution
Atg5	mouse	Santa Cruz Biotechnology, Santa Cruz, CA USA	1:100
Beclin-1	rabbit	Abcam	1:100
LC3A	rabbit	Sigma-Aldrich	1.25
LC3B	rabbit	Novus Biologicals	1:1000
p62	rabbit	Novus Biologicals	1:1000

Table S3. Correlation between LC3A methylation and clinicopathological parameters in primary lung tumor

		LC3A		p-value
		unmethylated	methylated	
**Type**	ADC	11	14	0.275
	SCC	9	22	
**Gender**	Male	18	28	0.749
	Female	4	9	
**Age**	< 65	14	24	1.000
	≥ 65	8	13	
**pN**	0-1	10	17	1.000
	>1	11	18	
**pT**	0-1	18	30	0.722
	>1	4	5	
**Grade**	1-2	14	20	0.589
	>2	8	17	

ADC: adenocarcinoma; SCC: squamous cell carcinoma.

Table S4. Correlation between p62 and clinicopathological data

		p62		p-value
		low	high	
**Type**	ADC	16	6	0.329
	SCC	52	35	
**Gender**	Male	62	40	0.405
	Female	12	4	
**pT**	0-1	54	37	0.182
	>1	20	7	
**pN**	0-1	68	35	0.084
	>1	6	9	
**Grade**	1-2	42	30	0.246
	>2	32	14	

Table S5. Correlation between Atg5 and clinicopathological data

		Atg5		p-value
		low	high	
**Type**	ADC	17	22	0.235
	SCC	38	30	
**Gender**	Male	49	57	0.873
	Female	12	13	
**Age**	≤ 65	37	47	0.313
	> 65	23	20	
**pT**	0-1	16	21	0.633
	≥ 2	45	49	
**pN**	0-1	16	21	0.633
	>1	45	49	
**pM**	0-1	53	67	0.112
	>1	8	3	
**Grade**	1-2	26	41	0.069
	>2	35	29	

ADC, adenocarcinoma; SCC, squamous cell carcinoma.
